# Deciphering the Molecular Mechanism of Flares in Patients with Systemic Lupus Erythematosus through Single-Cell Transcriptome Analysis of the Peripheral Blood

**DOI:** 10.31138/mjr.33.1.94

**Published:** 2022-03-31

**Authors:** Sofia Papanikolaou, Despoina Kosmara, Chrysoula Stathopoulou, Prodromos Sidiropoulos, Dimitrios Konstantopoulos, George Bertsias

**Affiliations:** 1 Rheumatology and Clinical Immunology, University of Crete Medical School and University Hospital of Heraklion, Heraklion, Greece,; 2 Infections and Immunity, Institute of Molecular Biology and Biotechnology, Foundation for Research and Technology – Hellas (FORTH), Heraklion, Greece,; 3 Single Cell Analysis Unit, Biomedical Sciences Research Center “Alexander Fleming”, Vari, Greece

**Keywords:** systemic lupus erythematosus, flares, genomics, epigenetic

## Abstract

A remarkable, yet poorly explained feature of Systemic Lupus Erythematosus (SLE) is the propensity to flare following a preceding period of disease inactivity. The clinical burden of lupus flares is substantial since they often tend to involve multiple or major organs, and carry a near two-fold increased risk for accrual of irreversible organ damage. The cellular and molecular mechanisms underlying the progression of SLE from inactive to active state remain ill-defined. Application of novel sequencing technologies together with cellular immunology assays, have illustrated the important role of multiple types of both innate and adaptive cells and associated pathways. We have previously described significant differences in the blood transcriptome of SLE patients at active versus inactive disease, and we have also defined genome regions (domains) with co-ordinated expression of genes implicated in the disease. In the present study, we aim to decipher the cellular and molecular basis of SLE exacerbations by utilising novel single-cell sequencing approaches, which allow us to characterise the transcriptional and epigenetic landscapes of thousands of cells in the peripheral blood of patients. The significance of the study lies in the detailed characterisation of the molecular and regulatory program of immune cell subpopulations that underlie progression from inactive to active SLE. Accordingly, our results may be exploited to identify biomarkers for disease monitoring and novel therapeutic targets.

## BACKGROUND AND STUDY RATIONALE

One of the intriguing characteristics of Systemic Lupus Erythematosus (SLE) is its tendency for increases in disease activity, also known as flares, which are often unpredictable. Indeed, in various patient cohorts, the frequency of SLE flares (or relapses) is approximately 0.30–0.50 per patient-year, and despite available treatments, long-term disease quiescence occurs only in a small fraction of patients.^1^ The clinical burden of lupus flares is remarkable, since about 30–40% of exacerbations affect multiple or major organs (for instance, kidneys), they necessitate treatment escalation or switch (including administration of high-dose glucocorticoids or other potent immunosuppressives), and are linked to almost 2-fold increased risk for irreversible organ damage.^2,3^ Importantly, treatment of SLE relapses remains empirical due to lack of randomised evidence and personalised options based on the underlying pathophysiology.

The cellular and molecular mechanisms implicated in SLE re-activation following a previous period of quiescence remain elusive.^4^ During recent decades, the advent of next-generation genome sequencing technologies coupled with focused cellular immunology studies, have provided unique insights regarding the role of specific cell types of both innate (eg, neutrophils) and adaptive (eg, follicular helper T-cells, long-lived plasmablasts) immunity as well as of pertinent pathways (eg, interferon-alpha, autoantibodies) in SLE pathogenesis.^5–10^

To this end, the gene expression and functional state of cells may be altered under the effect of exogenous or other (for instance, inflammatory cytokines) factors through epigenetic changes including histone and chromatin modifications.^11,12^ In fact, changes in the epigenetic and transcriptional program of tumour-surveilling immune cells have been linked to resistance to chemotherapy and progression of malignant disease.^13,14^

In a previous work using RNA-sequencing (RNA-seq), we identified widespread perturbations in gene expression in the peripheral blood of active SLE patients as compared to their counterparts at remission or low disease activity state.^15^ A total of 693 genes showed differential expression in patients with active versus inactive or low disease activity, and these genes were enriched in pathways such as type I interferon, proteasome, and oxidative phosphorylation. Further analysis revealed that genomes of SLE patients are organised into distinct regions of co-expressed genes (known as *Domains of Coordinated gene Expression [DCEs]*), which seem to regulate important pathways, such as interferon.^16^ These DCEs varied according to the level of SLE activity and correlated with changes in chromatin accessibility. Notably, the genomes of patients at low disease activity or remission displayed co-expression of genes implicated in kidney disease and neutrophil activation, which suggests the persistence of genomic aberrations contributing to disease flare-up.

Furthermore, other research groups have described alterations in chromatin accessibility and/or activation of gene enhancers in SLE patient-derived monocytes,^17,18^ neutrophils^19^ and B-cells.^20,21^ Altogether, these data underscore a possible role of the chromatin environment and epigenetic factors on determining the transcriptional program and function of immune cells contributing to lupus. Still, the time-dynamics of the aforementioned molecular cues and the interaction between various immune cell types have not been studied in the context of SLE transition from inactive to active state.

## AIMS OF THE STUDY

In this research study, we aim to investigate the cellular and molecular basis of flares in patients with SLE. Considering the complexity of the disease, we will utilise state-of-the-art single-cell sequencing technologies to obtain a detailed map of the transcriptional and epigenetic profiles in multiple subtypes of peripheral blood immune cells. Through a prospective biosampling protocol, we plan to simultaneously characterise gene expression and chromatin accessibility at single-cell resolution in the peripheral blood mononuclear cells (at least 3,000 to 5,000 cells per patient sample) from two (2) SLE patients assayed at three (3) consecutive time points: disease remission, flare and post-flare (after treatment modification). Our hypothesis is that specific epigenetic modifications (chromatin accessibility) direct the molecular reprogramming of specific immune cells thus contributing to SLE relapse.

## METHODS

This is a prospective, non-interventional, clinical-translational study with the following design and implementation plan.

### Patient recruitment

Patients diagnosed with SLE will be screened consecutively and recruited from the Connective Tissue Disease out-patient clinic of the Rheumatology Department, University Hospital of Heraklion, Greece. Inclusion criteria will include: a) fulfilment of the 2019 EULAR/ACR (European Alliance of Associations for Rheumatology / American College of Rheumatology) classification criteria,^22^ b) age 18–40 years, c) history of positive anti-dsDNA autoantibodies, d) relapsing-remitting disease pattern,^23^ and e) low disease activity or remission during the past 3 months according to the LLDAS^24^ and DORIS25 definitions, respectively. Exclusion criteria will include: a) coexistence of other systemic autoimmune or inflammatory disease, b) pregnancy or planning for pregnancy, c) chronic infection, d) history of malignancy, e) treatment with cyclophosphamide during the previous 3 months or with rituximab during the previous 6 months. The study will receive approval by the Institutional Review Boards of the University Hospital of Heraklion and the University of Crete, and all participants will provide informed consent form.

### Sample size

Considering that single-cell technologies enable the profiling of thousands of individual cells (up to 10,000 cells per sample) from peripheral blood samples, a relatively small number of patients is adequate. Since this is a pilot study, only two (2) patients will be analysed, each patient sampled on three consecutive time points: during remission or low disease activity, at the time of flare, 3 months post-flare and after treatment administration.

Patient samples of each time point will be combined and profiled in a separate 10x Multiome library. To identify two eligible patients, a total 20 SLE patients who meet the aforementioned inclusion criteria will be monitored clinically over a period of 12 months.

### Clinical monitoring and bio-sampling protocol

Enrolled patients will be monitored every 3–4 months for a total period of 12 months. On each visit, routine laboratory, serological (C3/C4, anti-dsDNA) and urine tests will be performed, and disease activity will be quantified by the validated indices: a) SLE Disease Activity Index-2000 (SLEDAI-2K)^26^ and b) Physician Global Assessment (PhGA; scale 0–3).^27^ For the detection and quantification of flares, the SELENA-SLEDAI Flare Index (SFI) will be employed,^27^ following minor modifications as outlined in **Table 1**. Patient history, physical examination and any relevant laboratory or imaging tests will be considered to exclude any flare-mimics such as infection or drug adverse effect. Administered medications and dosages will be recorded.

**Table 1.** Modified SELENA-SLEDAI Flare Index.^27^

**Moderate flare**	**Severe flare**
Increase in SLEDAI by ≥3 points (but not to >12)	Increase in SLEDAI to >12 points
New or worse: discoid, photosensitive, profundus, cutaneous vasculitis or bullous lupus rash, nasopharyngeal ulcers, serositis, arthritis, fever	New/worse: neurological lupus, vasculitis, nephritis, myositis, platelet <60.000/μL, haemolytic anaemia (Hb <7 g/L or ↓ Hb > 3 g/L)
Increase or added prednisone, but to a dose <30 mg/day	Increase or added prednisone to a dose ≥30mg/day, or pulses of intravenous methylprednisolone
Added NSAID or antimalarials (for SLE activity)	Added cyclophosphamide, azathioprine, methotrexate or mycophenolate, or new biological drugs (rituximab, belimumab) for SLE activity, or hospitalization
Increase in PhGA by ≥1 (but not to >2.5)	Increase in PhGA to >2.5

SLEDAI, SLE disease activity index; NSAID, nonsteroid anti-inflammatory drugs; PhGA, physician global assessment; Hb, haemoglobin.

Upon inclusion to the study, all patients will donate a first blood sample (15 ml) obtained by venipuncture (low disease activity/remission stage). During follow-up, a second blood sample will be obtained from two SLE patients who will develop moderate or severe flare according to the SFI (flare stage). Those patients will be managed for active disease at the discretion of the treating physician and in line with the EULAR recommendations.^28^ Three months after therapeutic intervention of the flare, a third blood sample will be collected (post-flare stage).

### Isolation of peripheral blood mononuclear cells and single-cell RNA-seq/ATAC-seq

We will assay blood as a relevant and easily accessible tissue to define complex inflammatory signatures.^29,30^ Venous samples (2 patients x 3 time-points, 6 in total) will undergo Ficoll density centrifugation for isolation of peripheral blood mononuclear cells (PBMCs). In SLE, the PBMCs fraction also includes circulating neutrophils,^31^ which are relevant to disease pathogenesis. Single-cell suspensions will be prepared on microplates through the robotic Fluidigm C1 system, followed by purification of RNA and chromatin from the cell nuclei.^32^ Genomic material will be processed for library synthesis (DNA for chromatin, cDNA for gene expression) and analysis by the chromium-based (10x) single-cell multiome ATAC (Assay for Transposase-Accessible Chromatin) & gene expression.^32–34^ Next-generation sequencing will be carried out at the Genomic Facility, IMBB-FORTH.

### Bioinformatics and data analysis

We will create a single-cell transcriptomic and epigenomic atlas followed by modularity optimisation to detect immune cell clusters across patient groups and time-points.^35,36^ Differential gene expression between pertinent immune cell clusters, coupled with functional enrichment analysis, will characterise the cellular states and dedicated transcriptional programs predisposing to flares. Chromatin accessibility data will help to disentangle the regulatory programs governing the transition to active SLE, guided by the cell-to-cell linkage between modalities (scRNA-seq, scATAC-seq) and the cell-state annotations of the transcriptome atlas. Using the scATAC-seq data, we will characterise our immune cell clusters, and determine high resolution cell sub-clusters with unique chromatin accessibility profiles. Open chromatin regions (OCRs) will be subjected to functional enrichment analysis (LOLA, GREAT tools) to infer their putative regulatory roles.^37,38^ Also, mapping of transcription factor (TF) binding sites (TFBS) motifs^39^ will reveal enrichment of specific TFs involved in the acquisition of a primed state of immune cells. Correlation between gene expression, TF expression and motif accessibility will identify flares-predisposing Gene Regulatory Networks (TF-to-gene promoter/enhancer relationships). Finally, reconstruction of immune cell lineage differentiation and cell maturation trajectories will delineate the cellular dynamics and underlying states during SLE re-activation*.*^35,40^ Collectively, our analysis will decipher how gene regulatory reprogramming in immune cells triggers flare initiation. We will also test the reversibility of these molecular/regulatory programs in the post-flare samples.

## ANTICIPATED RESULTS AND PROJECT SIGNIFICANCE

Patients with SLE often manifest flares (relapses) of their disease, which contribute to substantial clinical, financial,^41^ and societal burden and tend to trigger treatment intensification including administration of glucocorticoids. The cellular and molecular mechanisms underlying transition of SLE from inactive to active state remain incompletely understood. In view of the biological complexity of the disease, we hereby propose the utilisation of novel, high-resolution genomic technologies which enable the simultaneous profiling of gene expression and chromatin state (accessibility) in circulating blood cells of the lupus patients both during disease quiescence, flare-up, and following introduction of therapy. Through this approach, a detailed atlas of the genome will be obtained in thousands of cells assayed across consecutive time points. We will be able to characterise specific immune cell sub-populations that are altered in terms of frequency and/or functional state during SLE flare. In addition, chromatin analysis will provide novel insights into regulatory/epi-genetic drivers of perturbed transcriptomes associated with disease re-activation. Altogether, our analysis could uncover novel and biologically-relevant biomarkers for disease remission and exacerbation, as well as putative targetable genes and pathways for flare prevention and treatment.

## References

[1] Adamichou C Bertsias G. Flares in systemic lupus erythematosus: diagnosis, risk factors and preventive strategies. Mediterr J Rheumatol 2017;28(1):4–12. 32185248 10.31138/mjr.28.1.4 PMC7045928

[2] Stoll T Sutcliffe N Mach J Klaghofer R Isenberg DA. Analysis of the relationship between disease activity and damage in patients with systemic lupus erythematosus--a 5-yr prospective study. Rheumatology (Oxford) 2004;43(8):1039–44. 15161983 10.1093/rheumatology/keh238

[3] Ugarte-Gil MF Acevedo-Vasquez E Alarcon GS Pastor-Asurza CA Alfaro-Lozano JL Cucho-Venegas JM The number of flares patients experience impacts on damage accrual in systemic lupus erythematosus: data from a multiethnic Latin American cohort. Ann Rheum Dis 2015;74(6):1019–23. 24525909 10.1136/annrheumdis-2013-204620

[4] Fernandez D Kirou KA. What Causes Lupus Flares? Curr Rheumatol Rep 2016;18(3):14. 26951252 10.1007/s11926-016-0562-3

[5] Catalina MD Owen KA Labonte AC Grammer AC Lipsky PE. The pathogenesis of systemic lupus erythematosus: Harnessing big data to understand the molecular basis of lupus. J Autoimmun 2020;110:102359. 31806421 10.1016/j.jaut.2019.102359

[6] Frangou E Georgakis S Bertsias G. Update on the cellular and molecular aspects of lupus nephritis. Clin Immunol 2020;216:108445. 32344016 10.1016/j.clim.2020.108445

[7] Fritsch RD Shen X Illei GG Yarboro CH Prussin C Hathcock KS Abnormal differentiation of memory T cells in systemic lupus erythematosus. Arthritis Rheum 2006;54(7):2184–97. 16802356 10.1002/art.21943

[8] Rose T Dorner T. Drivers of the immunopathogenesis in systemic lupus erythematosus. Best Pract Res Clin Rheumatol 2017;31(3):321–33. 29224674 10.1016/j.berh.2017.09.007

[9] Tsokos GC. Autoimmunity and organ damage in systemic lupus erythematosus. Nat Immunol 2020;21(6):605–14. 32367037 10.1038/s41590-020-0677-6 PMC8135909

[10] Tsokos GC Lo MS Costa Reis P Sullivan KE. New insights into the immunopathogenesis of systemic lupus erythematosus. Nat Rev Rheumatol 2016;12(12):716–30. 27872476 10.1038/nrrheum.2016.186

[11] Divangahi M Aaby P Khader SA Barreiro LB Bekkering S Chavakis T Trained immunity, tolerance, priming and differentiation: distinct immunological processes. Nat Immunol 2021;22(1):2–6. 33293712 10.1038/s41590-020-00845-6 PMC8020292

[12] van der Heijden C Noz MP Joosten LAB Netea MG Riksen NP Keating ST. Epigenetics and Trained Immunity. Antioxid Redox Signal 2018;29(11):1023–40. 28978221 10.1089/ars.2017.7310 PMC6121175

[13] Bi M Zhang Z Jiang YZ Xue P Wang H Lai Z Enhancer reprogramming driven by high-order assemblies of transcription factors promotes phenotypic plasticity and breast cancer endocrine resistance. Nat Cell Biol 2020;22(6):701–15. 32424275 10.1038/s41556-020-0514-z PMC7737911

[14] Cohen YC Zada M Wang SY Bornstein C David E Moshe A Identification of resistance pathways and therapeutic targets in relapsed multiple myeloma patients through single-cell sequencing. Nat Med 2021;27(3):491–503. 33619369 10.1038/s41591-021-01232-w PMC7612793

[15] Panousis NI Bertsias GK Ongen H Gergianaki I Tektonidou MG Trachana M Combined genetic and transcriptome analysis of patients with SLE: distinct, targetable signatures for susceptibility and severity. Ann Rheum Dis 2019;78(8):1079–89. 31167757 10.1136/annrheumdis-2018-214379 PMC6691930

[16] Ntasis VF Panousis NI Tektonidou MG Dermitzakis ET Boumpas DT Bertsias GK Extensive fragmentation and re-organization of transcription in Systemic Lupus Erythematosus. Sci Rep 2020;10(1):16648. 33024230 10.1038/s41598-020-73654-4 PMC7539002

[17] Shi L Zhang Z Song L Leung YT Petri MA Sullivan KE. Monocyte enhancers are highly altered in systemic lupus erythematosus. Epigenomics 2015;7(6):921–35. 26442457 10.2217/epi.15.47 PMC4864065

[18] Park SH Kang K Giannopoulou E Qiao Y Kang K Kim G Type I interferons and the cytokine TNF cooperatively reprogram the macrophage epigenome to promote inflammatory activation. Nat Immunol 2017;18(10):1104–16. 28825701 10.1038/ni.3818 PMC5605457

[19] Mistry P Nakabo S O’Neil L Goel RR Jiang K Carmona-Rivera C Transcriptomic, epigenetic, and functional analyses implicate neutrophil diversity in the pathogenesis of systemic lupus erythematosus. Proc Natl Acad Sci U S A 2019;116(50):25222–8. 31754025 10.1073/pnas.1908576116 PMC6911190

[20] Scharer CD Blalock EL Barwick BG Haines RR Wei C ATAC-seq on biobanked specimens defines a unique chromatin accessibility structure in naive SLE B cells. Sci Rep 2016;6:27030. 27249108 10.1038/srep27030 PMC4888756

[21] Scharer CD Blalock EL Mi T Barwick BG Jenks SA Deguchi T Epigenetic programming underpins B cell dysfunction in human SLE. Nat Immunol 2019;20(8):1071–82. 31263277 10.1038/s41590-019-0419-9 PMC6642679

[22] Aringer M Costenbader K Daikh D Brinks R Mosca M Ramsey-Goldman R 2019 European League Against Rheumatism/American College of Rheumatology classification criteria for systemic lupus erythematosus. Ann Rheum Dis 2019;78(9):1151–9. 31383717 10.1136/annrheumdis-2018-214819

[23] Nikpour M Urowitz MB Ibanez D Gladman DD. Frequency and determinants of flare and persistently active disease in systemic lupus erythematosus. Arthritis Rheum 2009;61(9):1152–8. 19714602 10.1002/art.24741

[24] Franklyn K Lau CS Navarra SV Louthrenoo W Lateef A Hamijoyo L Definition and initial validation of a Lupus Low Disease Activity State (LLDAS). Ann Rheum Dis 2016;75(9):1615–21. 26458737 10.1136/annrheumdis-2015-207726

[25] van Vollenhoven RF Bertsias G Doria A Hanly J St-Pierre Y Gordon C 2021 DORIS definition of remission in SLE: final recommendations from an international task force. Lupus Sci Med 2021;8(1). 10.1136/lupus-2021-000538 PMC8614136 34819388

[26] Gladman DD Ibanez D Urowitz MB. Systemic lupus erythematosus disease activity index 2000. J Rheumatol 2002;29(2):288–91. 11838846

[27] Petri M Kim MY Kalunian KC Grossman J Hahn BH Sammaritano LR Combined oral contraceptives in women with systemic lupus erythematosus. N Engl J Med 2005;353(24):2550–8. 16354891 10.1056/NEJMoa051135

[28] Fanouriakis A Kostopoulou M Alunno A Aringer M Bajema I Boletis JN 2019 update of the EULAR recommendations for the management of systemic lupus erythematosus. Ann Rheum Dis 2019;78(6):736–45. 30926722 10.1136/annrheumdis-2019-215089

[29] Duffy D Rouilly V Libri V Hasan M Beitz B David M Functional analysis via standardized whole-blood stimulation systems defines the boundaries of a healthy immune response to complex stimuli. Immunity 2014;40(3):436–50. 24656047 10.1016/j.immuni.2014.03.002

[30] Urrutia A Duffy D Rouilly V Posseme C Djebali R Illanes G Standardized Whole-Blood Transcriptional Profiling Enables the Deconvolution of Complex Induced Immune Responses. Cell Rep 2016;16(10):2777–91. 27568558 10.1016/j.celrep.2016.08.011

[31] Villanueva E Yalavarthi S Berthier CC Hodgin JB Khandpur R Lin AM Netting neutrophils induce endothelial damage, infiltrate tissues, and expose immunostimulatory molecules in systemic lupus erythematosus. J Immunol 2011;187(1):538–52. 21613614 10.4049/jimmunol.1100450 PMC3119769

[32] See P Lum J Chen J Ginhoux F. A Single-Cell Sequencing Guide for Immunologists. Front Immunol 2018;9:2425. 30405621 10.3389/fimmu.2018.02425 PMC6205970

[33] Aissa AF Islam A Ariss MM Go CC Rader AE Conrardy RD Single-cell transcriptional changes associated with drug tolerance and response to combination therapies in cancer. Nat Commun 2021;12(1):1628. 33712615 10.1038/s41467-021-21884-z PMC7955121

[34] Haque A Engel J Teichmann SA Lonnberg T. A practical guide to single-cell RNA-sequencing for biomedical research and clinical applications. Genome Med 2017;9(1):75. 28821273 10.1186/s13073-017-0467-4 PMC5561556

[35] Cano-Gamez E Soskic B Roumeliotis TI So E Smyth DJ Baldrighi M Single-cell transcriptomics identifies an effectorness gradient shaping the response of CD4(+) T cells to cytokines. Nat Commun 2020;11(1):1801. 32286271 10.1038/s41467-020-15543-y PMC7156481

[36] Penkava F Velasco-Herrera MDC Young MD Yager N Nwosu LN Pratt AG Single-cell sequencing reveals clonal expansions of pro-inflammatory synovial CD8 T cells expressing tissue-homing receptors in psoriatic arthritis. Nat Commun 2020;11(1):4767. 32958743 10.1038/s41467-020-18513-6 PMC7505844

[37] Rendeiro AF Krausgruber T Fortelny N Zhao F Penz T Farlik M Chromatin mapping and single-cell immune profiling define the temporal dynamics of ibrutinib response in CLL. Nat Commun 2020;11(1):577. 31996669 10.1038/s41467-019-14081-6 PMC6989523

[38] Yan F Powell DR Curtis DJ Wong NC. From reads to insight: a hitchhiker’s guide to ATAC-seq data analysis. Genome Biol 2020;21(1):22. 32014034 10.1186/s13059-020-1929-3 PMC6996192

[39] Boeva V. Analysis of Genomic Sequence Motifs for Deciphering Transcription Factor Binding and Transcriptional Regulation in Eukaryotic Cells. Front Genet 2016;7:24. 26941778 10.3389/fgene.2016.00024 PMC4763482

[40] Campbell KR Yau C. Uncovering pseudotemporal trajectories with covariates from single cell and bulk expression data. Nat Commun 2018;9(1):2442. 29934517 10.1038/s41467-018-04696-6 PMC6015076

[41] Bertsias G Karampli E Sidiropoulos P Gergianaki I Drosos A Sakkas L Clinical and financial burden of active lupus in Greece: a nationwide study. Lupus 2016;25(12):1385–94. 27055520 10.1177/0961203316642310

